# *Lactobacillus acidophilus* Endocarditis Complicated by Pauci-Immune Necrotizing Glomerulonephritis

**DOI:** 10.1155/2020/1607141

**Published:** 2020-08-11

**Authors:** Vivian O. Chukwurah, Comfort Takang, Chinelo Uche, David B. Thomas, Waguih El Masry, Hakan R. Toka

**Affiliations:** ^1^Graduate Medical Education, Manatee Memorial Hospital, Bradenton, FL, USA; ^2^Manatee Kidney Diseases Consultants, Bradenton, FL, USA

## Abstract

Infective endocarditis (IE) is more common in patients with predisposing cardiac lesions and has many potential complications, including stroke and arterial thromboembolisms. Renal manifestations have an estimated prevalence of ∼20%. Rapidly progressive glomerulonephritis (RPGN) is a nephrological emergency manifested by autoimmune-mediated progressive loss of renal function over a relatively short period of time. Here, we report the case of a 60-year-old Caucasian male, who presented with speech impairment and was found to have multiple embolic strokes caused by aortic valve IE. His renal function declined rapidly, and his urine sediment featured hematuria and proteinuria. ANCA titer was negative by immunofluorescence (IF); however, the PR3 antibody was elevated. The renal biopsy revealed pauci-immune focally necrotizing glomerulonephritis with the presence of ∼25% cellular crescents. He was initially treated with plasmapheresis and pulse dose steroids. Hemodialysis was initiated for uremic symptoms. After four weeks of antibiotic therapy and with blood cultures remaining negative, he was treated with rituximab. Two months after discharge, his renal function showed improvement, and hemodialysis was discontinued. This case highlights several complications associated with lactobacillus endocarditis including RPGN.

## 1. Introduction

Lactobacillus is a Gram-positive, microaerophilic rod-shaped bacterium that is a part of the normal flora in oral, gastrointestinal, and urogenital cavities. It has been associated with dental carries, urinary tract infections, and to a much lesser frequency with splenic abscesses [1, 2]. *Lactobacillus acidophilus* is a rare cause of bacteremia and, very rarely, infective endocarditis (IE). Documented clinical features range from asymptomatic to severe septicemia. Lactobacillus bacteremia is thought to be underdiagnosed as a lot of cultures are dismissed as contaminants.

Renal disease is a well-known complication of IE and is included in the Duke's criteria for IE. Rapidly progressive glomerulonephritis (RPGN) is an umbrella term used in this context to include ANCA-associated pauci-immune necrotizing and crescentic glomerulonephritis (GN), anti-GBM disease, and immune complex-mediated kidney injuries. GN syndromes are often recognized by the presence of hematuria and proteinuria [3]. Furthermore, RPGN presents with severe acute kidney injury in a relatively short period of time, ranging from days to weeks, and if not treated, it frequently progresses to end-stage renal disease [4].

## 2. Case Presentation

A 60-year-old Caucasian male presented with acute onset of confusion and expressive aphasia. The patient was not able to recognize his wife and appeared clumsy. He did not have a previous history of confusion, mental illness, or illicit drug use. His prior medical history consisted of controlled hypertension, non-insulin-dependent diabetes mellitus type 2, and chronic lower back pain, for which he used ibuprofen as needed.

**Figure 1:**
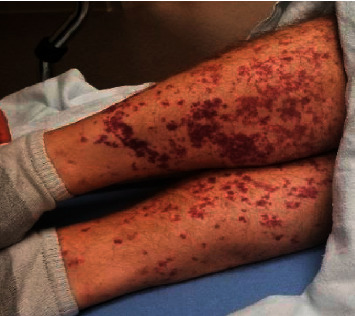
Purpuric rash on bilateral lower extremities.

**Figure 2:**
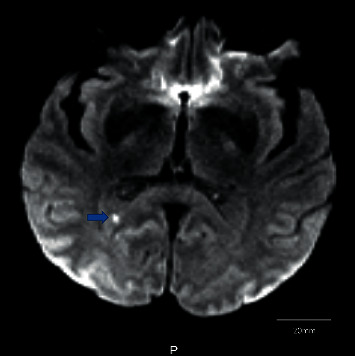
MRI of brain with contrast showing acute-to-subacute 5.3 mm embolic brain infarct (blue arrow).

On presentation, he was afebrile; blood pressure was 141/91 mmHg; heart rate, 91/min; respiratory rate, 16/min; and oxygen saturation, 99% on room air. On evaluation, he was a well-built male, pleasantly confused, and unable to respond appropriately to orientation questions. Physical findings included dental carries on the right and left lower molars. On cardiac exam, there were no audible murmurs, rubs, gallops, or ectopy. Lungs were clear on auscultation, and his abdomen was nondistended and nontender. His lower extremities showed bilateral nonblanching, palpable purpuric lesions, primarily involving his shins (Figure 1). On neurological exam, cranial nerves II–XII were grossly intact. He had decreased motor strength with 3/5 on his right lower extremity; his sensation was intact, and Babinski exam was negative. His deep tendon reflexes were 2+ bilaterally, and finger-to-nose was intact. He had expressive aphasia, but intact repetition. Dyscalculia, dysarthria, dysphonia, or dysgraphia were not noted. Differential diagnosis at the time of presentation included acute stroke, bacteremia, and toxic or metabolic encephalopathy. His brain MRI noted an 8 mm left parietal lobe acute-to-subacute infarct (Figure 2).

**Figure 3:**
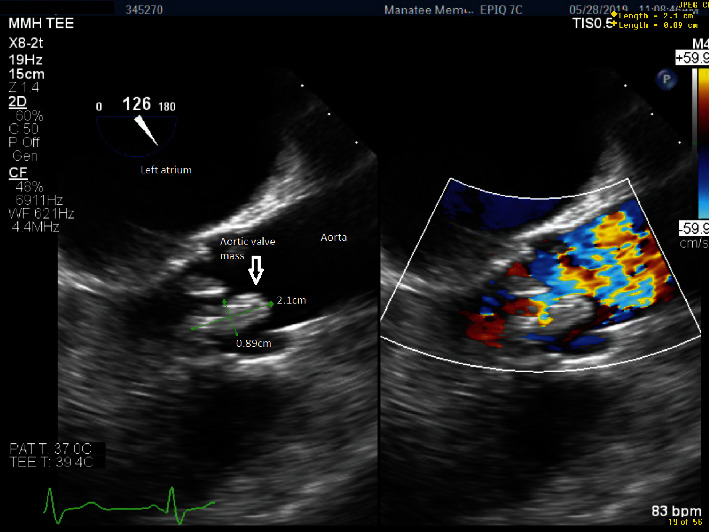
TEE: long-axis view of the aortic valve shows partially calcified aortic leaflet vegetation with significant aortic regurgitation per Doppler study.

During the hospital course, the patient developed recurrent episodes of low-grade fevers with *T*-max ∼100.8 F, progressively worsening mentation and generalized weakness. Lumbar puncture was negative for viral, bacterial, or fungal infections. A repeat brain MRI noted two new areas of acute ischemia, ∼3 mm in left parietal white matter and the posterior corpus callosum. On cardiac exam, auscultation revealed a loud midsystolic peaking murmur on the right upper sternal border. Suspecting IE, broad spectrum antibiotic therapy was initiated. Blood cultures obtained upon admission were reported to be positive for *Lactobacillus acidophilus*. The subsequent transesophageal echocardiogram (TEE) showed a partially calcified aortic valve with severe aortic regurgitation and mild aortic stenosis (Figure 3). The patient's serum creatinine continued to rise rapidly. His urine sediment showed muddy brown casts consistent with acute tubular necrosis and dysmorphic RBCs. Serological workup revealed an elevated PR3 (proteinase 3) antibody titer of 16.3 (reference range: 0–3.5), while p-ANCA (<1 : 20) and c-ANCA (<1 : 20) titers were negative by immunofixation electrophoresis (IFE).

**Figure 4:**
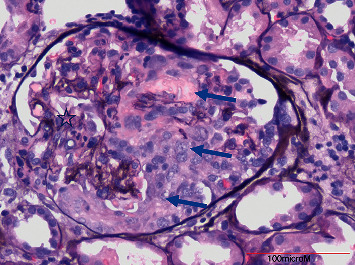
Light microscopy featuring an exemplary glomerulus with collapsed capillaries (star) and extensive cellular crescents (blue arrows). Stain performed with periodic acid methamine silver (PAMS) (original magnification 20x).

The patient was started on intravenous pulse steroids, 1 gm solumedrol daily for three days, followed by 60 mg of prednisone daily. He was started on plasmapheresis and had a total of four sessions. The renal biopsy showed 22 glomeruli per tissue section, four of these showing segmental fibrinoid necrosis, segmental karyorrhectic debris, and cellular crescents.  Figure 4 features light microscopy of an exemplary glomerulus featuring cellular crescents. No glomerular endocapillary hypercellularity was identified. Mild interstitial fibrosis with tissue atrophy was noted to be ∼10%. Patchy slight interstitial edema and mononuclear interstitial inflammation were present. The immunofluorescence was negative, and the diagnosis of pauci-immune focal necrotizing glomerulonephritis was made. After completion of four weeks intravenous antibiotic therapy with piperacillin/tazobactam, two doses of rituximab, 1000 mg, two weeks apart, were administered. The patient was given trimethoprim/sulfamethoxazole for pneumocystis carinii prophylaxis and continued steroid taper for a total of four months. Upon completion of steroid therapy, he underwent a successful aortic valve replacement surgery. His kidney function improved two months after initiation of steroids to the level of no longer requiring dialysis. His serum creatinine level remains stable at 1.6 mg/dl three months after cardiac surgery and is consistent with CKD stage 3. His urine analysis remains bland without presence of hematuria or proteinuria, and repeat anti-PR3 Ab testing was negative.

## 3. Discussion

The initial clinical presentation of this case, expressive aphasia and confusion, is unusual for infectious endocarditis (IE). The brain MRI showed embolic brain foci and as our patient developed fever, the diagnosis of endocarditis emerged. Lactobacillus bacteremia and an aortic valve vegetation seen on echocardiogram established the diagnosis of IE. The typical signs and symptoms of IE, fever, heart murmur, Janeway lesions, splinter hemorrhage, and Osler nodes [5], were initially not present. Although uncommon, acute cerebrovascular accidents as initial presentation of aortic valve endocarditis have been reported previously [6].

Also noteworthy are the rarity and high mortality associated with lactobacillus endocarditis. As of 2005, there were only 78 reported cases dating back to 1992 [7]. *Lactobacillus acidophilus* is mostly found in the mouth, gastrointestinal tract, and vagina as part of normal flora [8]. The causality of our patient's lactobacillus endocarditis was initially questionable given that he denied IV drug use, recent GI surgery, colonoscopy, probiotic use, oral sex, or dental procedures, but was eventually attributable to his dental carries. Though not very common, lactobacillus bacteremia has been reported to result from dental carries [9, 10]. It is possible that the chronic use of ibuprofen in our patient could have resulted in erosion of the lining of the GI tract, increasing the likelihood of the entry of lactobacillus bacteria (originating from dental carries) into the blood stream. His infection was primarily managed with piperacillin/tazobactam, although there are limited data on treatment and susceptibility of lactobacillus [7]. He received a six-week course of IV antibiotics including piperacillin/tazobactam (weeks 1–4), ceftriaxone (only week 2), and ertapenem (weeks 5 and 6) per our infectious disease team recommendations and recommended guidelines for treatment duration of native valve IE [11].

The differential diagnosis for an acute kidney injury (AKI) is often broad, including prerenal, intrinsic, and postrenal etiologies. Initially, we suspected renal hypoperfusion as causes of his AKI based on his NSAID use and may be poor oral fluid intake. Due to the absence of pyuria or proteinuria, acute interstitial nephritis or minimal change disease were considered unlikely. When the patient developed muddy brown casts, the diagnosis of ATN was made. Microscopic examination of his urine identified dysmorphic RBCs, suggesting a vasculitic process. The presence of palpable purpura on his lower extremities was also consistent with vasculitis. The serologic workup was positive for anti-PR3, while ANCA screen by immunofluorescence was negative. Although a false-positive Ab titer could be considered, this is very unlikely due to high specificity of PR3 antibodies for vasculitis. The fine needle kidney biopsy confirmed pauci-immune focal necrotizing GN with cellular crescents [12].

GN is a well-documented complication of IE; however, ANCA-negative RPGN is uncommon [13]. Due to the severity of his AKI, we decided to treat with aggressive immunosuppressive therapy. The patient received rituximab, two doses of 1000 mg administered two weeks apart per recommended guidelines [14]. Rituximab is a chimeric monoclonal antibody against protein CD20, which is primarily found on the surface of B lymphocytes. It is an effective treatment for granulomatosis with polyangiitis [14]. Due to rituximab-induced B-cell depletion, we delayed treatment until the patient completed four weeks of antibiotic therapy. Plasmapheresis was discontinued based on the interpretation of the recent PEXIVAS trial data, which reported no benefit of plasma exchange for renal outcomes in severe ANCA-associated crescentic glomerulonephritis [15]. Similarly, steroids were tapered within a four-month period as lower steroid dosing and duration seeming equally effective per PEXIVAS.

Despite the high mortality rate of ∼30% for lactobacillus endocarditis [16], our case had a positive outcome and recovered. There are no established treatment guidelines for ANCA vasculitis in IE. The decision of immunosuppressive treatment is made on case-to-case basis and can include steroids, plasmapheresis, and aggressive immunosuppressants [17]. This case suggests that rituximab may have a role in the treatment of IE-associated GN, in addition to antibiotics and steroids, and could be considered in aggressive disease.

Our patient continues to follow with his nephrologist since his aortic valve replacement. His kidney function remains stable, blood pressure is controlled on low-dose ACE inhibitor, his urine analysis is bland, and his anti-PR3 is negative following the completion of steroid therapy. A follow-up plan for acute GN due to IE has not been established; however, we suggest monitoring (a) blood pressure routinely, (b) BUN and serum creatinine levels every 3 months for 1 year (then, to follow KDIGO guidelines for CKD), and (c) check urine for hematuria every 3 months for at least 1 year. Measuring routinely PR3 Ab titers is not recommended unless patient would show signs or symptoms of recurring GN.
